# Gene sequence analysis model construction based on k-mer statistics

**DOI:** 10.1371/journal.pone.0306480

**Published:** 2024-09-12

**Authors:** Dongjie Gao

**Affiliations:** School of Mathematics and Statistics, Heze University, Heze, China; King Faisal University, SAUDI ARABIA

## Abstract

With the rapid development of biotechnology, gene sequencing methods are gradually improved. The structure of gene sequences is also more complex. However, the traditional sequence alignment method is difficult to deal with the complex gene sequence alignment work. In order to improve the efficiency of gene sequence analysis, D2 series method of k-mer statistics is selected to build the model of gene sequence alignment analysis. According to the structure of the foreground sequence, the sequence to be aligned can be cut by different lengths and divided into multiple subsequences. Finally, according to the selected subsequences, the maximum dissimilarity in the alignment results is determined as the statistical result. At the same time, the research also designed an application system for the sequence alignment analysis of the model. The experimental results showed that the statistical power of the sequence alignment analysis model was directly proportional to the sequence coverage and cutting length, and inversely proportional to the K value and module length. At the same time, the model was applied to the system designed in this paper. The maximum storage capacity of the system was 71 GB, the maximum disk capacity was 135 GB, and the running time was less than 2.0s. Therefore, the k-mer statistic sequence alignment model and system proposed in this study have considerable application value in gene alignment analysis.

## 1. Introduction

With the development of high-throughput sequencing technology, genomics research has entered a new era. The analysis of genome sequences has become an important part of biological and medical research [1]. The analysis of genome sequence can reveal the function of genes, the characteristics of genome structure, and the association between genomes, which is of great significance for the in-depth understanding of biological activities and the mechanism of disease. In the process of genome sequence analysis, the method based on k-mer statistics has attracted researchers’ attention. K-mer refers to continuous subsequences with a length of K. Abundant sequence information can be obtained by counting the frequency of different k-mers in genome sequences [2]. K-mer statistics can be used in many fields such as feature extraction, sequence similarity comparison, genome classification and prediction of genome sequences. However, due to the complexity of k-mer statistics and the relatively simple research depth, there are still few studies on the application of k-mer statistics in the alignment analysis of long gene sequences [3]. In order to promote the application of k-mer statistics in gene sequence alignment analysis, this study takes the D_2_ series of methods in k-mer statistics as the research object, innovatively constructs a sequence statistical model, and determines a unified measure for it, so as to realize the frequency identity and visualization of sequence data. At the same time, in order to apply the constructed sequence statistical model to the actual sequence analysis, the research also designed the sequence alignment software, and applied the model to the software, aiming to achieve an efficient sequence alignment function. To complete the above research content, the structure of the article is divided into five parts. The first part is a brief introduction to the research content of the article. The second part is the development status of the research direction. The third part is divided into two sections, which respectively expounds the operation principle of gene sequence and k-mer statistics, as well as the model and system constructed by using k-mer statistics. The fourth part is the experimental analysis part, which sets up different experiments to analyze the performance of the model and system. The fifth part is the summary of the research content. The purpose of this paper is to promote the application prospect of k-mer statistics in gene sequence alignment.

## 2. Related works

In biomedicine, statistics are widely used. Statistical methods play a crucial role in analyzing and interpreting biological data, enabling researchers to gain insights and understand the trends and significance of the data. Additionally, these methods facilitate data visualization, allowing for a more intuitive and efficient understanding of the information by researchers. Y. Fan et al used statistical methods to analyze the DNA integrity of cancer patients. The study set up a control experiment, which divided cancer patients into two groups before and after surgery. The statistical results showed that the DNA integrity of patients after operation was better than that before operation, and there was no statistical difference between the two groups. Therefore, the statistical analysis of DNA integrity can be used for the diagnosis of lung cancer disease [4]. C. Randler et al. used statistical methods to investigate and analyze the phenomenon of bird tail flick. After investigating and recording the behavior of birds in different environments, they used statistical methods to analyze the causes of bird tail flicks. The results showed that the tail flick phenomenon was mainly affected by the predation risk [5]. L. Crawford et al. used the statistical method to statistically analyze the biological information in GBM images. Through the analysis of quantitative statistical methods, the results showed that the gene expression and volume characteristics of GBM could better reflect the status of GBM patients. Therefore, the results of statistical analysis can be used for the diagnosis of GBM disease [6]. X. Yin et al. used statistical methods to analyze 100 loci of systemic lupus erythematosus. Firstly, according to the pathological conditions of different systemic lupus erythematosus cases, the human genetic region was determined, and then the 28 association signals were analyzed by Bayesian statistics. Finally, the genetic association signals of systemic lupus erythematosus loci were analyzed according to the statistical probability results. A total of 10 loci with posterior probability ≥ 0.8 were selected [7].

In the field of biology, gene sequence carries most of the biological genetic information, so the measurement and analysis of gene sequence has very important research value. In order to evaluate the functionality of precise cloning, J. Ludwig et al. obtained the gene sequence information in the TCR gene library. This study set up a high-throughput analysis tool, which can link TCR gene sequence and cell phenotype at the cellular level and perform functional analysis. It is believed that this method can realize the analysis of the mouse TCR gene [8]. L. Wu et al. studied the mechanism of self-incompatibility of Petunia by gene sequence analysis. The comparative analysis of S-site sequences of three Petunia S haplotypes, it revealed that there was a potential genetic exchange in the flanking region of the Petunia S gene, which promoted the self-incompatibility of Petunia [9]. J. Sérgio et al. found the pathogen of the virus by sequencing the complete genome of dogs infected with canine leptospirosis and computer analysis and completely inferred the variation process of the virus. The analysis results showed that the 56609 serotype strain was genetically related to the virus [10]. In order to study the drug resistance of the HIV-1 gene, T. O. Digban et al. performed gene sequencing analysis on patients with the HIV-1 disease and used the Mega 6 inference phylogenetic analysis tool. The results of the study showed that in the gene comparison, no mutation of the drug-resistance gene was found, so the traditional drug administration treatment scheme can still be used [11]. In order to determine the mediating relationship between DNA sequence and protein, M. Menzel et al. used high-throughput sequencing to measure the DNA sequence and protein sequence and determined the binding sites of the two sequences by k-mer statistics [12].

In conclusion, in the field of biology, statistical methods have achieved high application value in the process of application. It can not only intuitively reflect the association and change trend between biological data, but also provide researchers with a visual data scheme. Because gene sequences contain a lot of genetic information, sequence alignment, and analysis can promote the development of biology and other related technologies. Although k-mer statistic method has high statistical performance, few researchers apply it to gene sequence analysis. The data inclusiveness of k-mer statistic method is very suitable for the alignment analysis of long gene sequences. Therefore, this study uses the k-mer statistic method to build the gene sequence alignment model, aiming to improve the application value of the k-mer statistic method in gene sequence alignment analysis.

## 3 Gene sequence alignment model construction and application based on k-mer statistics

With the rapid development of gene technology, the measured gene sequence has more perfect information, so the length of the sequence has also increased significantly. Because most of the traditional sequence alignment methods have made it difficult to deal with gene sequence alignment for massive data, the k-mer statistic with high computational performance and high inclusiveness is proposed to carry out applied research on gene sequence alignment.

### 3.1 Principle research based on k-mer statistics and gene sequence

#### 3.1.1 Gene sequence data composition

**Fig 1:**
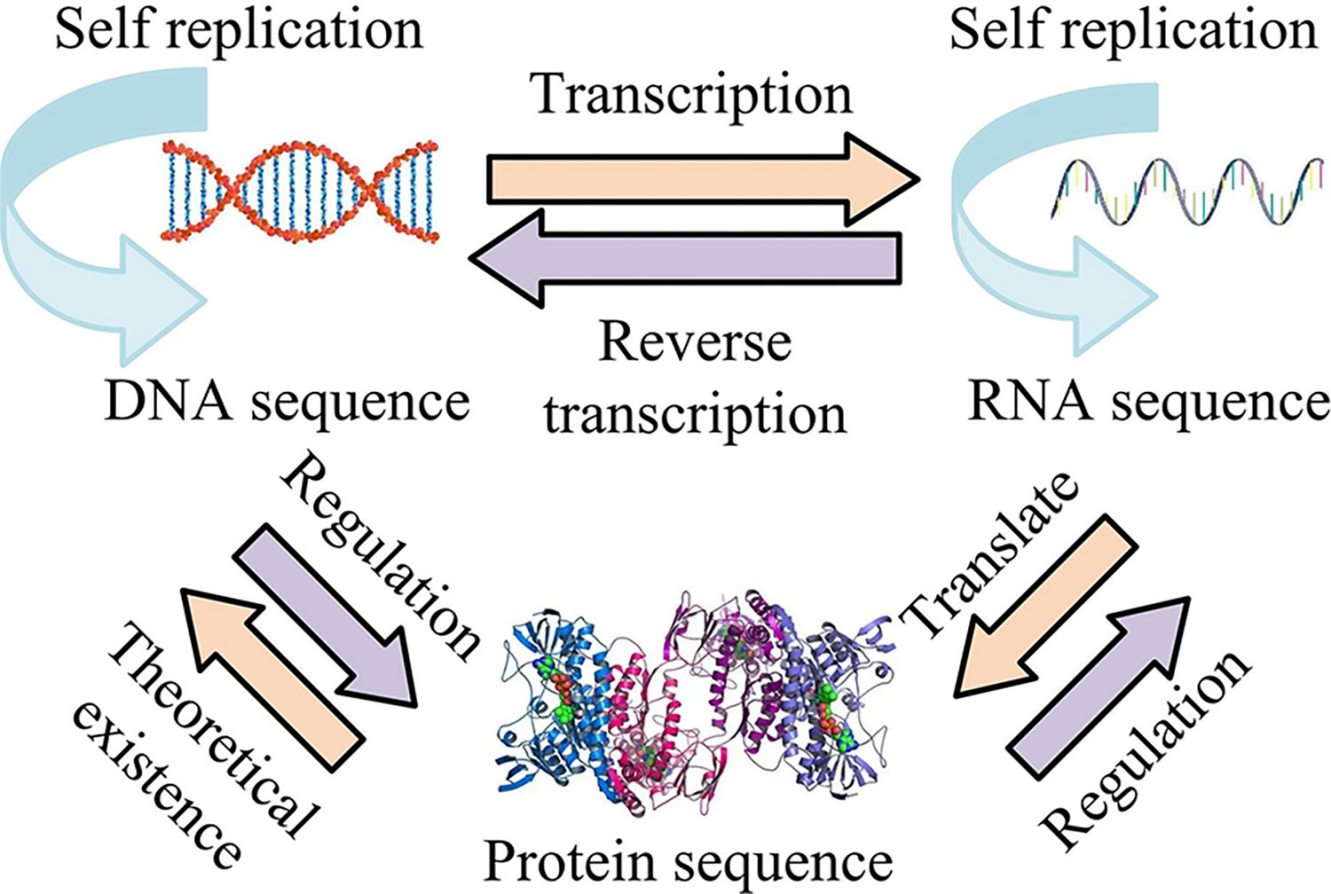
Transformation relationships between biological sequences.

Biological sequence refers to the linear sequence of biological molecules such as genome, transcriptome, and proteome in an organism. In biology, common biological sequences include deoxyribonucleic acid (DNA) and ribonucleotide (RNA) gene sequences and macromolecular protein sequences. Among them, the DNA sequence is composed of adenine (A), guanine (G), thymine (T), and cytosine (C), and the arrangement mainly relies on a series of base pairs pairing with each other [13]. The main arrangement of base pairs is A-T and G-C. The base composition of RNA sequence is different from that of DNA sequence. There is no thymine in the RNA sequence, but uracil (U) instead. The protein sequence is a linear arrangement consisting of 20 amino acids. There is a certain biological relationship between gene sequence and protein sequence, and the specific biological relationship is shown in Fig 1.

Fig 1 shows the transformation relationship between biological sequences. The biological relationships shown in Fig 1 are called the central law in biology. As the carrier of genetic information, DNA can replicate itself. Under the action of RNA polymerase, genetic information is transcribed into RNA through transcription factors. RNA can also realize the self-replication of genetic information and transmit genetic information to proteins by editing free RNAs, which is also a translation process. At the same time, in some viruses, RNA also has the function of reverse transcription of genetic information to DNA. Proteins can regulate DNA and RNA. Among them, both DNA sequence and RNA sequence are gene sequences in organisms.

DNA is a molecule that stores genetic information in an organism. The molecule is a double-stranded structure formed by two complementary single-stranded DNA through base pairing and winding together in the form of a helix. Among them, each single-stranded DNA is composed of a series of bases, which are interconnected by hydrogen bonds. The two single-stranded DNAs of double-stranded DNA are arranged in opposite directions, that is, the 5 ’end of one strand corresponds to the 3’ end of the other strand, while the 3 ’end of one strand corresponds to the 5’ end of the other strand. The gene sequence of DNA is randomly arranged by A, T, C, and G, such as in GCATTACG-CGTA form. RNA gene sequence refers to the sequence of RNA molecules that encode proteins in organisms. RNA is a single-stranded structure. The gene sequence of this molecule is the primary structure of RNA. It is mainly a random combination of A, U, C, and G to form the corresponding sequence, such as ACGCCG-GCUAGC.

In conclusion, DNA and RNA molecules contain a large amount of biological genetic information, and the analysis of their biological sequences will help biotechnologists to further explore the biological mechanism.

#### 3.1.2 Basic operation process of D2 statistic based on k-mer

The k-mer statistic is a method used to analyze DNA or RNA sequences. K-mer is a continuous subsequence of length k, where k is a positive integer. The k-mer statistic represents the number or frequency of occurrences of each different k-mer calculated in the sequence. The calculation of k-mer statistics can not only obtain the distribution of sequences but also reveal the similarities or differences between sequences by comparing the k-mer statistics of different sequences [14]. In practical applications, the commonly used K value is usually 4, 5, or 6, which can provide enough information to describe the sequence characteristics, and the calculation efficiency is high. By analyzing k-mer statistics, researchers can better understand and interpret the structure and function of biological sequences.

The *D*_2_ series statistics method of k-mer statistics is used in this study. It is assumed that there are gene sequences *X* and *Y*. Among them, the length of the sequence *X* is n characters, and the length of the sequence *Y* is m characters. The data set *A* = {*A*,*T*,*G*,*C*} is composed of sequences, and then the sequence k-mer length and 4^*k*^ k-mer species are calculated according to the *k* values. Assuming the existence of any k-mer species, it can be expressed as Eq (1).


$$w=w_{1}w_{2}\ldots w_{k}$$ (1)

In Eq (1), *w*_*k*_ is composed of the *k* k-mer statistic. Then the sequence *X* and *Y* can be expressed as *X*_*w*_ and *Y*_*w*_, that is, the k-mer vector can represent any gene sequence. Gene sequences can be aligned according to the *k* constituent elements in the k-mer vector. If the *D*_2_ statistic is used for comparison, the expression is shown in Eq (2).


$$D_{2}=\sum_{w\in A}X_{w}Y_{w}$$ (2)

In Eq (2), the number of k-mer vectors is mainly considered in the calculation of *D*_2_ statistics, while the total content of k-mer is ignored. Therefore, the traditional *D*_2_ statistical algorithm will be affected by the sequence length and noise. Therefore, *D*^*S*^_2_ and D*_2_ methods are proposed. Both *D*^*S*^_2_ and D*_2_ methods need to carry out central standardization treatment first, and the treatment equation is shown in Eq (3).


$$\left\{ \begin{array}{c} {\bar{X}}_{w}=X_{w}-(n-k+1){p^{X}}_{w}\\ {\bar{Y}}_{w}=X_{w}-(m-k+1){p^{Y}}_{w} \end{array}\right.$$ (3)

In Eq (3), *p*^*X*^_*w*_, *p*^*Y*^_*w*_, and *p*^*Y*^_*w*_ are the *w* occurrence probabilities in sequences *X* and *Y*, respectively. After normalizing the sequence, the calculation equation of the D^S^_2_ statistic is shown in Eq (4).


$${D^{S}}_{2}=\sum_{w\in A}\frac{{\bar{X}}_{w}{\bar{Y}}_{w}}{\sqrt{{{\bar{X}}_{w}}^{2}+{{\bar{Y}}_{w}}^{2}}}$$ (4)

The calculation equation of the D*_2_ statistic method is shown in Eq (5).


$${D^{*}}_{2}=\sum_{w\in A}\frac{{\bar{X}}_{w}{\bar{Y}}_{w}}{\sqrt{(n-k+1){p^{X}}_{w}(m-k+1){p^{Y}}_{w}}}$$ (5)

In Eq (5), the D*_2_ statistic uses Poisson distribution to approximate the variance of k-mer, but this method requires that the k-mer vector has a certain length. For comparative analysis of gene sequences using the *D*_2_ series statistics method, it is necessary to calculate the distance difference between sequences. The calculation equation of distance is shown in Eq (6).


{Eu=(∑w∈A|Xwn−Ywm|2)12Ma=∑w∈A|Xwn−Ywm|Ch=maxw∈A|Xwn−Ywm| (6)

In Eq (6), Eu, *Ma*, and *Ch* are the Euclidean distance, Manhattan distance, and Chebyshev distance between gene sequences, respectively. However, using distance to quantify the difference between sequences still needs a unified standard. The measure calculated by the three *D*_2_ methods is dissimilar. In biology, dissimilarity is usually used to measure the degree of difference between gene sequences, protein sequences, or organisms. The calculation methods of the three dissimilarities are shown in Eq (7).


$$\left\{ \begin{array}{c} d_{2}=\frac{1}{2}(1-\frac{\sum_{w\in A}X_{w}Y_{w}}{\sqrt{\sum_{w\in A}{X_{w}}^{2}}\sqrt{\sum_{w\in A}{Y_{w}}^{2}}})\\ d_{2}S=\frac{1}{2}(1-\frac{\sum_{w\in A}\frac{{\bar{X}}_{w}{\bar{Y}}_{w}}{\sqrt{{\bar{X}}_{w}^{2}+{\bar{Y}}_{w}^{2}}}}{\sqrt{\sum_{w\in A}{{\bar{X}}_{w}}^{2}/\sqrt{{{\bar{X}}_{w}}^{2}+{{\bar{Y}}_{w}}^{2}}}\sqrt{\sum_{w\in A}{{\bar{Y}}_{w}}^{2}/\sqrt{{{\bar{X}}_{w}}^{2}+{{\bar{Y}}_{w}}^{2}}}})\\ {d_{2}}^{*}=\frac{1}{2}(1-\frac{\sum_{w\in A}\frac{{\bar{X}}_{w}{\bar{Y}}_{w}}{\sqrt{(n-k+1)(m-k+1){p^{X}}_{w}{p^{Y}}_{w}}}}{\sqrt{\sum_{w\in A}{{\bar{X}}_{w}}^{2}/\left((n-k+1){p^{X}}_{w}\right)}\sqrt{\sum_{w\in A}{{\bar{Y}}_{w}}^{2}/\left((m-k+1){p^{Y}}_{w}\right)}}) \end{array}\right.$$ (7)

In Eq (7), *d*_2_,*d*_2_*S*, *d*_2_*and are the dissimilarity corresponding to *D*_2_, *D*^*S*^_2_, and D*_2_ respectively. At present, with the development of Biostatistics technology, *D*^*S*^_2_ and D*_2_ methods are more universal because they can normalize sequences. Therefore, in this study, *D*^*S*^_2_ and D*_2_ methods were used to build the statistical analysis model of gene sequence.

### 3.2 Gene sequence statistical model construction and application based on *D*^*S*^_2_ and D*_2_ methods

#### 3.2.1 Gene sequence statistical model construction based on *D*^*S*^_2_ and D*_2_ methods

Because *D*^*S*^_2_ and D*_2_ methods can perform standard processing on sequences, the above two methods can be used to build sequence statistical models *T*_*sum*_ and perform statistical analysis on gene sequences measured by next-generation sequencing (NGS).

**Fig 2:**
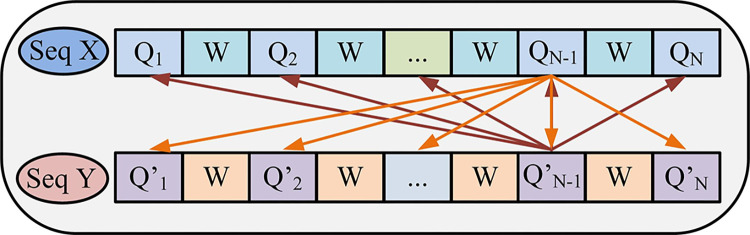
***T***_***sum***_ schematic diagram of statistical model operation.

Fig 2 is the schematic diagram of *T*_*sum*_ statistical model operation. Assuming that the target statistical sequences are *X* and *Y* this time, the model now cuts the two gene sequences that need comparative analysis separately and then extracts equal-length subsequences from the subsequences as statistical sequences. The sequence is cut from one end of the sequence, and then the sequence is cut with *Q* length, then w length, and then repeat the cutting sequence of *Q* and *W* length until the sequence is cut. The model only uses q-length subsequences as the statistical column and compares all Q-length subsequences with the complete sequence, and the statistical value is the maximum value of the comparison [15]. The expression for selecting the statistics of the process is shown in Eq (8).


$$\left\{ \begin{array}{c} {X^{s}}_{i}={max}_{1\leq i\leq N}{D_{2}}^{s}(W_{i}{{,W}^{\prime }}_{j})\\ {Y^{s}}_{j}={max}_{1\leq j\leq N}{D_{2}}^{s}(W_{i},{W^{\prime }}_{j}) \end{array}\right.$$ (8)

In Eq (8), *X*^*S*^_*i*_ and *Y*^*S*^_*j*_ are the statistical values of the *X* sequence and *Y* sequence respectively. After summing the subsequence statistical values of the *X* sequence and *Y* sequence respectively, and then multiplying, the sequence statistical values under the statistical model can be obtained. The *T*_*sum*_ statistical value of the sequence is shown in Eq (9).


$${T_{sum}}^{s}=\sum_{i=1}^{N}{X^{s}}_{i}\times \sum_{j=1}^{N}{Y^{s}}_{j}$$ (9)

**Fig 3:**
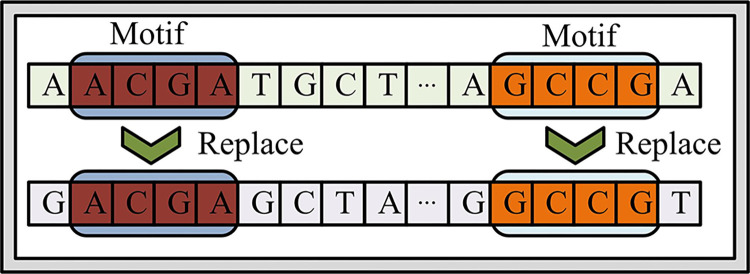
Schematic diagram of forest sequence model based on statistical power analysis.

In Eq (9), $\sum_{i=1}^{N}{X^{s}}_{i}$ and $\sum_{j=1}^{N}{Y^{s}}_{j}$ are the statistics of *X* subsequence and *Y* subsequence respectively. Since most of the sequences measured by NGS are fragment sequences, it is necessary to analyze the statistical power of the *T*_*sum*_ statistical model. In order to judge the efficacy of the *T*_*sum*_ statistical model studied, two DNA sequences with the same length were randomly generated by the computer. Among them, the base pair composition among sequences is the same distribution, that is, the probability of composition of a, t, C, and G is the same, which is 1 / 4. Then a generation model of the background sequence is constructed, and the foreground model is used to complete the power analysis of the*T*_*sum*_ statistical model [16]. The background sequence model for efficacy analysis is shown in Fig 3.

Fig 3 is a schematic diagram of the foreground sequence model based on statistical power analysis. The construction of the background sequence model is divided into two steps. First, the sequence sites with the same composition structure in the two sequences are randomly selected according to the Bernoulli distribution, and then the fragments with the composition structure are replaced with each other. The two sequences after replacement are foreground sequence models. After obtaining the model of the foreground sequence, the research can calculate the power of the *T*_*sum*_ statistical model. Assuming that the two sequences have high similarity when the significance level *t* reaches more than 95%, it shows statistical significance, and the hypothesis is true. If the significance level is less than 95%, the statistical model is not significant. While the model shows significance, it can also achieve high accuracy, that is, statistical power. The expression of statistical power is shown in Eq (10).


$$P=\frac{N_{Tsum\geq t}}{10000}$$ (10)

In Eq (10), *N*_*Tsum*_ is the foreground sequence with significance in the detection sample. 10000 is the number of statistics. *P* is the proportion of significant sequence. The value of *P* is [0,1]. The larger the value *P*, the stronger the statistical power of the model.

#### 3.2.2 Sequence statistical model application system design based on *D*_2_ series method

With the wide application of NGS technology in gene sequence detection, the integrity of the whole genome sequence gradually increases, resulting in the gradual increase of the data composition of the sequence. The traditional sequence alignment method represented by the systematic evolution method has made it difficult to deal with the massive data of complete NGS sequences. Therefore, the sequence non-alignment method has become a research hotspot. The sequence alignment method pays more attention to the analysis of the similarity between sequences, while the sequence non-alignment method will first convert the sequence into sub-sequences, calculate the dissimilarity between sub-sequences, determine the distance between sequences according to the dissimilarity, and finally establish the phylogenetic tree of sequences according to the calculated dissimilarity distance. Compared with the two, the sequence non-alignment method has higher data accommodation and objectivity [17]. The *T*_*sum*_ statistical model studied in this paper has excellent computational efficiency and is suitable for the application of NGS technology in multi-sequence non-alignment. In addition, the model has a parallel computing mode, which is conducive to the conversion calculation of different metrics in the process of sequence non-alignment. However, there are still controversies about the confirmation of standard metrics in sequence non-alignment methods, and there is also a lack of appropriate sequence analysis software as a tool to apply the method in practice. Therefore, it is necessary to develop a new system for comparative analysis of NGS sequences.

At present, *D*_2_ series methods are most widely used, so this study designs a SeqK system for sequence non-alignment based on the *d*_2_ dissimilarity of *D*_2_ series methods. The *d*_2_ metric can represent the k-mer count between two sequences and can sum all k-mers according to the determined *K* value. At the same time, *d*_2_-derived metric *d*_2_*S* and *d*_2_* can also be used in hierarchical gene sequences, and the non-aligned statistical results of sequences have high predictability.

**Fig 4:**
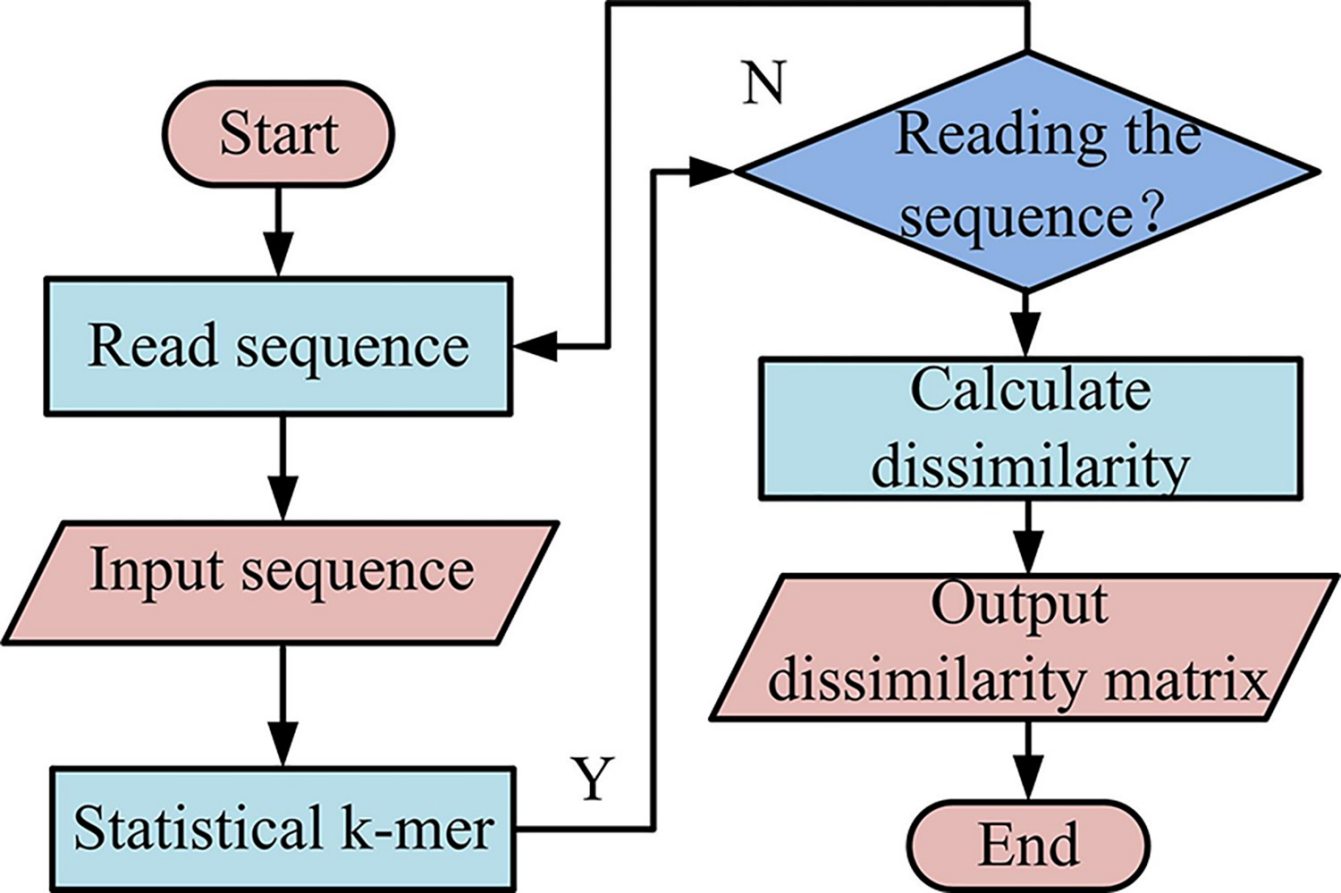
Calculation flowchart of SeqK system.

Fig 4 shows the calculation flow chart of the SeqK system. The SeqK system will first read a sequence randomly from all the input matrices and calculate the k-mer frequency in the sequence, then compare the two sequences according to the determined *d*_2_ metrics and calculate the dissimilarity matrix. According to the output dissimilarity matrix, the non-alignment of two sequences can be realized. Because the K-frequency in k-mer is relatively small, the comparison results of this method in the system are more accurate. Therefore, the research will also apply a method of controlling K-frequency in the SeqK system.

**Fig 5:**
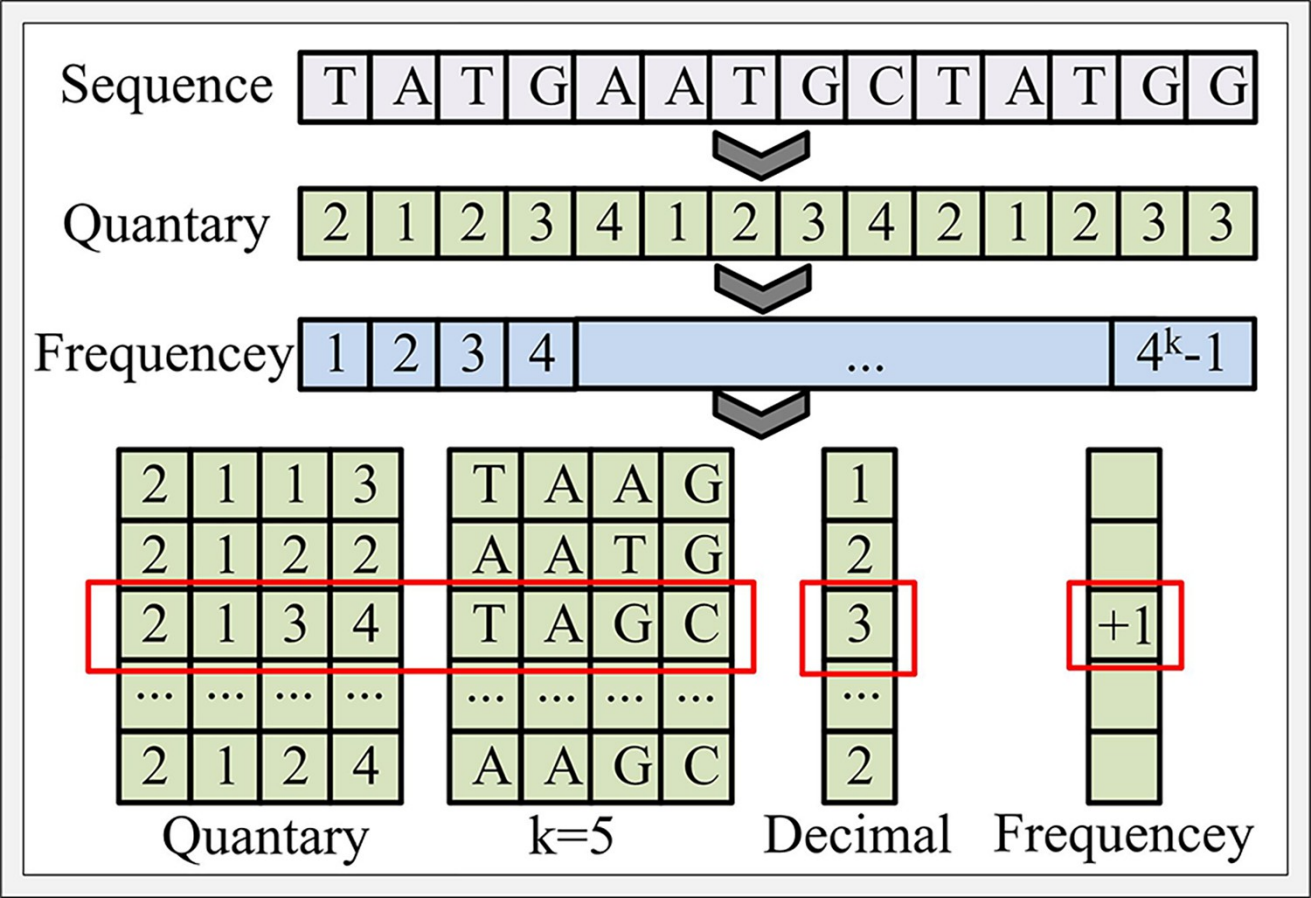
Calculation process diagram of K-frequency control algorithm.

Fig 5 shows the operation process of the K-frequency control algorithm. Because the data of gene sequence is composed of four letter structures, in order to facilitate the calculation of K-frequency, the sequence can first be converted into quaternary data, and then the k-mer name can be converted into a numerical index of the same base. The data index can control sequence addressing and data memory [18]. Then the k-mer frequency of the input sequence is substituted into the 4K vector. If there are N-aligned sequences, the vectors of the sequences are combined in a 4^*k*^×*N* matrix. Finally, according to the calculation method in Fig 4, the dissimilarity between different sequences is calculated and stored in the matrix. According to the obtained optimized dissimilarity matrix, the sequence can be analyzed.

## 4 Gene sequence analysis model performance and system application analysis based on k-mer statistics

In order to promote the application of k-mer statistics in the analysis of gene sequence alignment, this paper constructs a *T*_*sum*_ statistical model and designs a SeqK system, aiming to apply it to the system to realize the alignment of gene sequences. Therefore, in order to verify the performance of the model and system, different experiments were set up to verify it.

### 4.1 Experimental environment

In order to study the performance of k-mer statistics in gene sequence analysis, the SeqK system was designed to count k-mer vectors in sequences. The information on the hardware experiment environment implemented by the SeqK system is shown in Table 1.

**Table 1:** Experimental hardware environment.

Hardware Name	Information Type	Model Number
Server	Manufacturer	Intel
	Model Number	SYS-5038K-I-ES1
Processor	Model Number	Intel(R) Xeon Phi(TM) CPU 7210 @
	Number of CPUs	1
	Number of Cores	64
	Logical CPU	256
Memory	Manufacturer	Micron
	Combination	16GB×6
	Size	256GB
	Type	DDR4×6
Disk	Model	HGST HUS726040AL
	Size	4TB

Table 1 shows the experimental hardware environment table. At this time, the experiment uses the Intel server and Intel (R) Xeon Phi (TM) CPU 7210 @ server to build the SeqK system for experiments. The software information of the system is shown in Table 2.

**Table 2:** Experimental software sheet.

Software name	Software version	Version parameters
OS	Release	Ubuntu 20.04.1 LTS
	Kernel version	5.4.0-66-generic
Programming language	Standard	C++
Compiler	Version	GCC-9.3.0
	Optimization options	Fully optimized
	Architecture options	-march = X86-64
	Other options	-pthread-MCX16
Cuckoo hash table	Library name	libcuckoo
	Version	3.1.0

Table 2 shows the experimental software environment. The system used C++ language and the optimization compiler to display the sequence alignment of the system. According to the experimental environment in Table 1 and Table 2, the SeqK system can be applied to the computer platform, and the analysis performance of k-mer statistics in gene sequences can be explored by setting experiments.

### 4.2 Experimental setup

The original gene sequencing data from the National Center for Biotechnology Information (NCBI) were used in the experiment, and the data were measured by NGS technology. After breaking up the above gene sequences, the sequences were re-spliced by gene recombination technology to increase the length of the sequences. The recombined dataset information is shown in Table 3.

**Table 3:** Recombinant gene table.

Data Type	Homo sapiens	Triticum urartu	Tupaia chinensis	Capsicum annuum
Species Type	Humans	Monocotyledons	Tree shrews	Bilobed plants
Code	GRCh38.p12	Tu2.0	TupChi_1.0	Zunla 1
Sample number	GCF_000001405.38	GCA_003073215.1	GCF_000334495.1	GCF_000710875.1
Sequence type	DNA sequence	DNA sequence	DNA sequence	DNA sequence

Table 3 shows the list of recombinant genes. The gene types studied are mainly divided into four categories: human, monocot, tree shrew, and dicot. All four types of gene sequences were DNA sequences. The above sequences were divided into *Q* length and *W* length, and the sum of *Q* and *W* length and T represent the degree of fragmentation of the sequence. The smaller the *T*, the higher the degree of fragmentation of the sequence. In addition to sequence length *T* affecting the efficacy of the *T*_*sum*_ model, the coverage *r* of the model also had a certain impact. Coverage *r* represents the proportion of cut subsequences in all statistical sequences. Therefore, this study analyzed the *T*_*sum*_ statistical power under the influence of the module length *L* and frequency *k* of the foreground sequence.

### 4.3 *T*_*sum*_ model performance analysis based on k-mer statistics

The performance analysis of the statistical power of the *T*_*sum*_ model was expressed in the form of a statistical power *P* curve. The sequence gradient under the curve was selected in the span range of 500 to 2000. Each value was simulated 5,000 times and finally averaged. Firstly, the statistical power analysis of coverage under different sequence sets was carried out. The fragment length of the sequence was set to 2000, the frequency *k* to 6, and the module length *L* to 8. The effect of coverage on statistical power was analyzed according to the change in the p-value curve. Then the influence of k-mer frequency on the statistical power of the model was analyzed. The module length was set to 8, the coverage was 75%, and the coverage was set to 2000. The frequency ranged from 5, 6, 7 and 8. The effect on statistical power was determined according to the change in the p-value curve. Then the effect of sequence cut length on statistical power was analyzed. The frequency was set to 6, the module length to 8, the coverage rate to 75%, and the values to 500, 1000, 1500, and 2000. The effect on statistical power was determined according to the change in the p-value curve. Finally, the influence of module length on statistical power was analyzed. The frequency was set to 6, the coverage was 75%, and the value range was 5, 6, 7, and 8. The effect on statistical power was determined according to the change in the *p*-value curve. According to the above four groups of experiments, the statistical power of the *T*_*sum*_ model under different parameters was determined, and its performance was analyzed.

**Fig 6:**
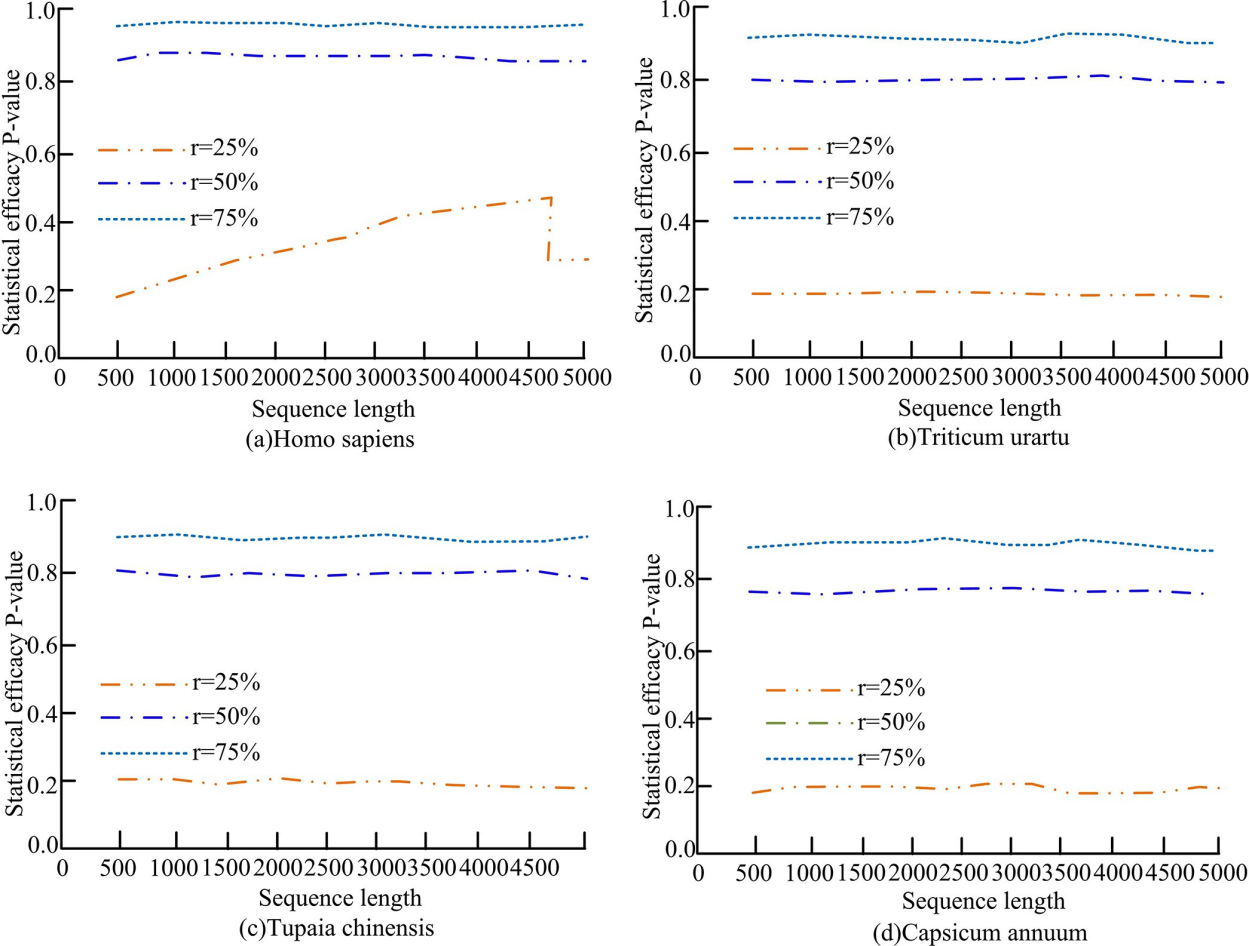
Statistical efficiency of *T*_*sum*_ models with different coverage in different sets of sequences.

Fig 6 shows the statistical power of models with different coverage in different sequence sets. Among them, Fig 6(A)–6(D) respectively use human, monocot, tree shrew and dicot sequence datasets. It can be seen that in the four datasets, the coverage rate was consistent with the statistical power of the model, and the statistical power of the model increased gradually with the increase of *r*. However, the length of the processing sequence had little effect on the statistical power of the model. When the coverage rate was high, the model processed sequence data more comprehensively, thereby improving the statistical power of the model. In four sequence datasets, when *r* was 25%, the statistical power of the model ranged from 0.17 to 0.30, which was relatively low. When *r* was 50%, the statistical power of the model ranged from 0.75 to 0.83. However, when *r* reached 75%, the statistical power of the model reached a range of 0.87–0.95. Therefore, the higher the coverage of the model, the higher the statistical power of the model.

**Fig 7:**
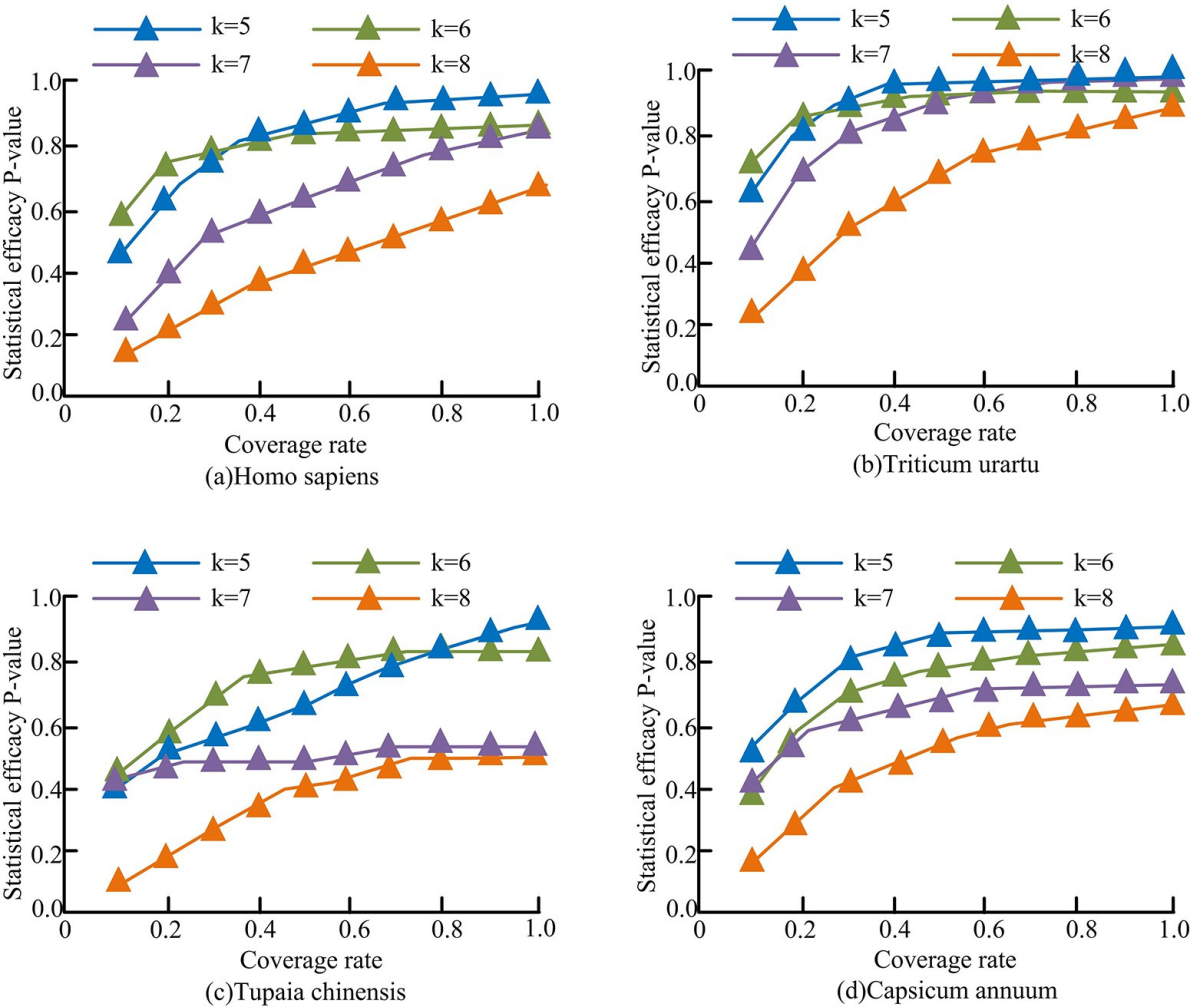
Statistical efficiency of *T*_*sum*_ models with different frequency in different sets of sequences.

Fig 7 shows the statistical power of models with different frequencies in different sequence sets. Among them, Fig 7(A)–7(D) respectively use human, monocot, tree shrew and dicot sequence datasets. It can be seen that in different sequence datasets, the changing trend of the statistical power of the model under different frequencies had certain differences. However, with the increase in coverage, the model with a smaller frequency can eventually achieve higher statistical power. Moreover, the overall statistical power of the model at different frequencies also increased. When *k* was 5 and the coverage was 1.0, the statistical power of the model reached 0.89, 0.93, 0.86, and 0.84 in the four sequence datasets of human, monocot, tree shrew, and dicot, respectively. When *k* was 6 and the coverage rate *r* was 1.0, the statistical power of the *T*_*sum*_ model reached 0.82, 0.85, 0.78, and 0.82 in the four sequence datasets of human, single leaf plant, tree shrew, and double leaf plant, respectively. When *k* was 7 and the coverage rate *r* was 1.0, the statistical power of the *T*_*sum*_ model reached 0.82, 0.79, 0.41, and 0.68 in the four sequence datasets of human, monocotyledonous, tree shrew, and dicotyledonous plants, respectively. When *k* was 8 and the coverage rate *r* was 1.0, the statistical power of the *T*_*sum*_ model reached 0.59, 0.78, 0.38, and 0.57 in the four sequence datasets of humans, monocotyledonous plants, tree shrews, and dicotyledonous plants, respectively. Therefore, the model under low frequency can achieve better statistical power.

**Fig 8:**
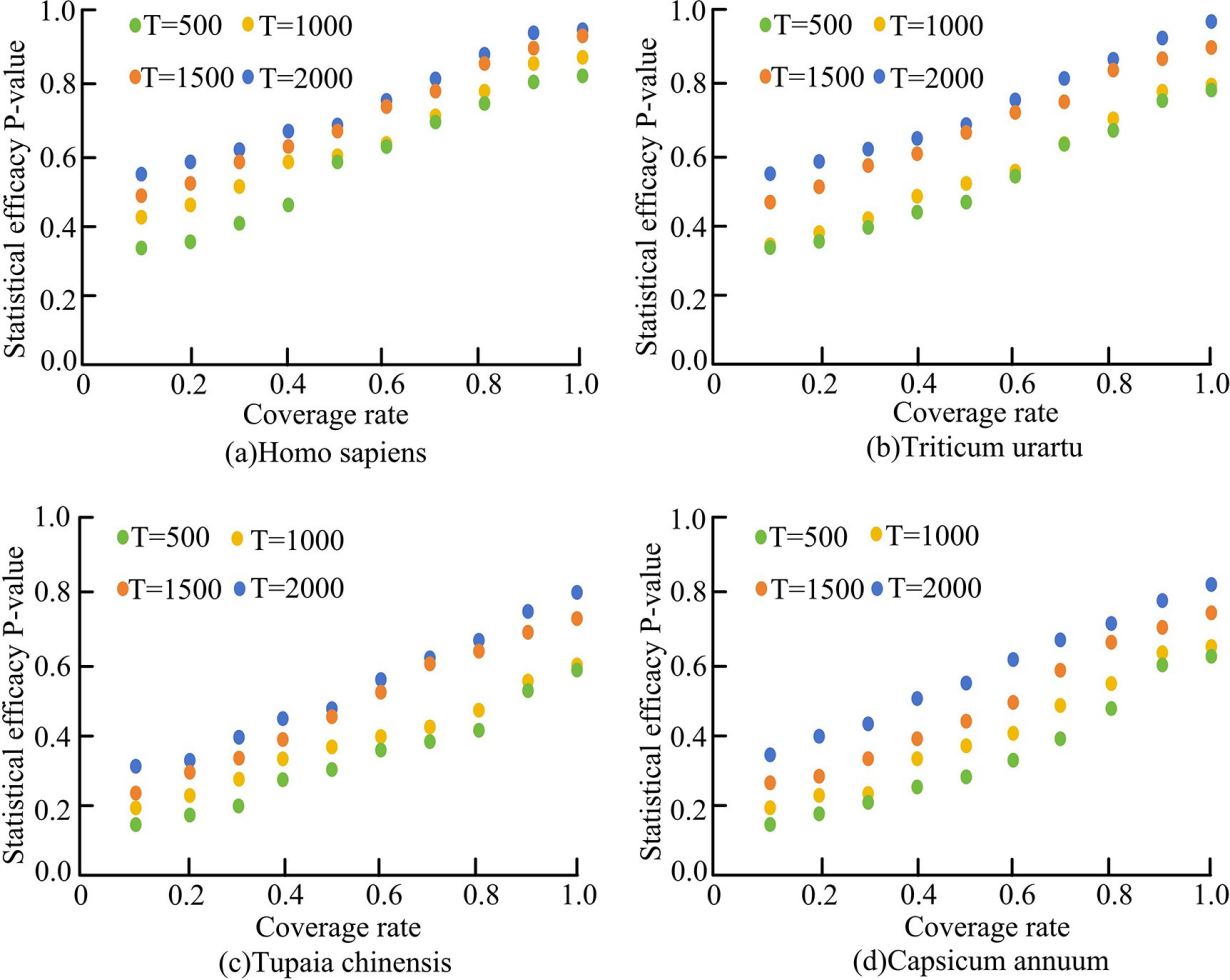
Statistical efficiency of *T*_*sum*_ models with different cut lengths in different sets of sequences.

Fig 8 shows the statistical power of models with different cutting lengths in different sequence sets. Among them, Fig 8(A)–8(D) respectively use human, monocot, tree shrew and dicot sequence datasets. It can be seen that in the four types of sequence data sets, the variation trend of the statistical efficiency of the model was basically the same. With the increase in sequence cutting length, the statistical power of the models showed an upward trend. Because the shorter the length of the cut subsequence, the higher the degree of fragmentation of the sequence. At this time, the model was more prone to appear similar modules in the alignment between sequences, which led to the decline of the statistical power of the model. When *T* was 500, the model *T*_*sum*_ achieved statistical efficacy of 0.72, 0.68, 0.52, and 0.52 in four sequence datasets of humans, monocotyledonous plants, tree shrews, and dicotyledonous plants, respectively. When *T* was 1000, the model *T*_*sum*_ achieved statistical efficacy of 0.76, 0.67, 0.53, and 0.54 in four sequence datasets of humans, monocotyledonous plants, tree shrews, and dicotyledonous plants, respectively. When *T* was 1500, the model *T*_*sum*_ achieved statistical power of 0.84, 0.82, 0.75, and 0.74 in four sequence datasets of humans, monocotyledonous plants, tree shrews, and dicotyledonous plants, respectively. When *T* was 2000, the model can achieve the statistical power of 0.87, 0.93, 0.80, and 0.79 in four sequence datasets of human, monocot, tree shrew, and dicot, respectively. Therefore, with the increase of the sequence cutting length, the statistical power of the model gradually increased.

**Fig 9:**
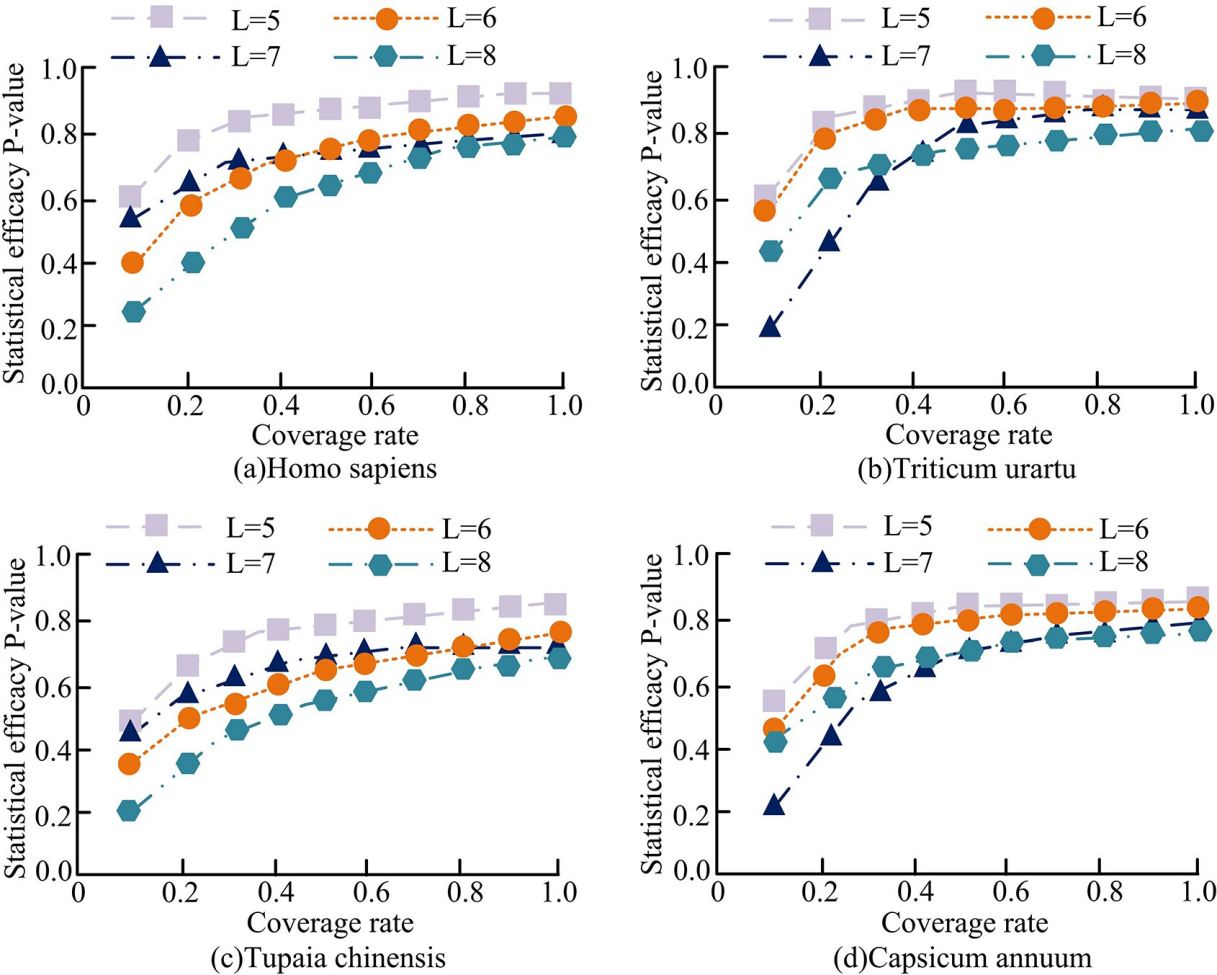
Statistical efficiency of *T*_*sum*_ models with different module lengths in different sets of sequences.

Fig 9 shows the statistical power of models with different module lengths in different sequence sets. Among them, Fig 9(A)–9(D) respectively use human, monocot, tree shrew and dicot sequence datasets. It can be seen that in different types of gene sequences, the model was negatively correlated with module length. As the module length decreased, the statistical power of the model gradually increased. When *L* was 5, the power of the model in human, monocot, tree shrew, and dicot sequence datasets can reach 0.80, 0.82, 0.81, and 0.81, respectively. When *L* was 6, the *T*_*sum*_ model achieved efficacy of 0.78, 0.81, 0.73, and 0.76 in four sequence datasets of humans, monocotyledonous plants, tree shrews, and dicotyledonous plants, respectively. When *L* was 7, the *T*_*sum*_ model achieved efficacy of 0.71, 0.72, 0.71, and 0.73 in four sequence datasets of humans, monocotyledonous plants, tree shrews, and dicotyledonous plants, respectively. When *L* was 8, the *T*_*sum*_ model achieved efficacy of 0.70, 0.70, 0.59, and 0.62 in four sequence datasets of humans, monocotyledonous plants, tree shrews, and dicotyledonous plants, respectively. Therefore, as the module length decreases, the statistical power of the model will gradually rise.

### 4.4 Sequence non-aligned SeqK system analysis based on k-mer statistics

In order to make the k-mer statistic method be applied to sequence alignment analysis, this study also designed the SeqK system, which quantifies the character composition structure in the gene sequence through the *D*_2_ series method in the k-mer statistic, so as to carry out the alignment analysis between series according to the dissimilarity matrix. In order to verify the application performance of the system in sequence alignment analysis, three classical sequence comparison systems, danman, basic local alignment search tool (BLAST), and Clustal, were used for analysis in this study. Among them, danman supports multiple sequence alignment, and the operation is relatively simple. Clustal is a multi-sequence alignment tool for progressive alignment, which is widely used. Blast is the most recognized tool in short sequence contrast, and the system has the advantage of high efficiency. The computing environments of the four systems are shown in Table 1 and Table 2. In the comparison of system performance, data sets with sequence lengths of 500, 1000, 1500, and 2000 were set up, and the four sets of data were input into the system respectively to analyze the operation time and hardware operation state of different computing systems.

**Fig 10:**
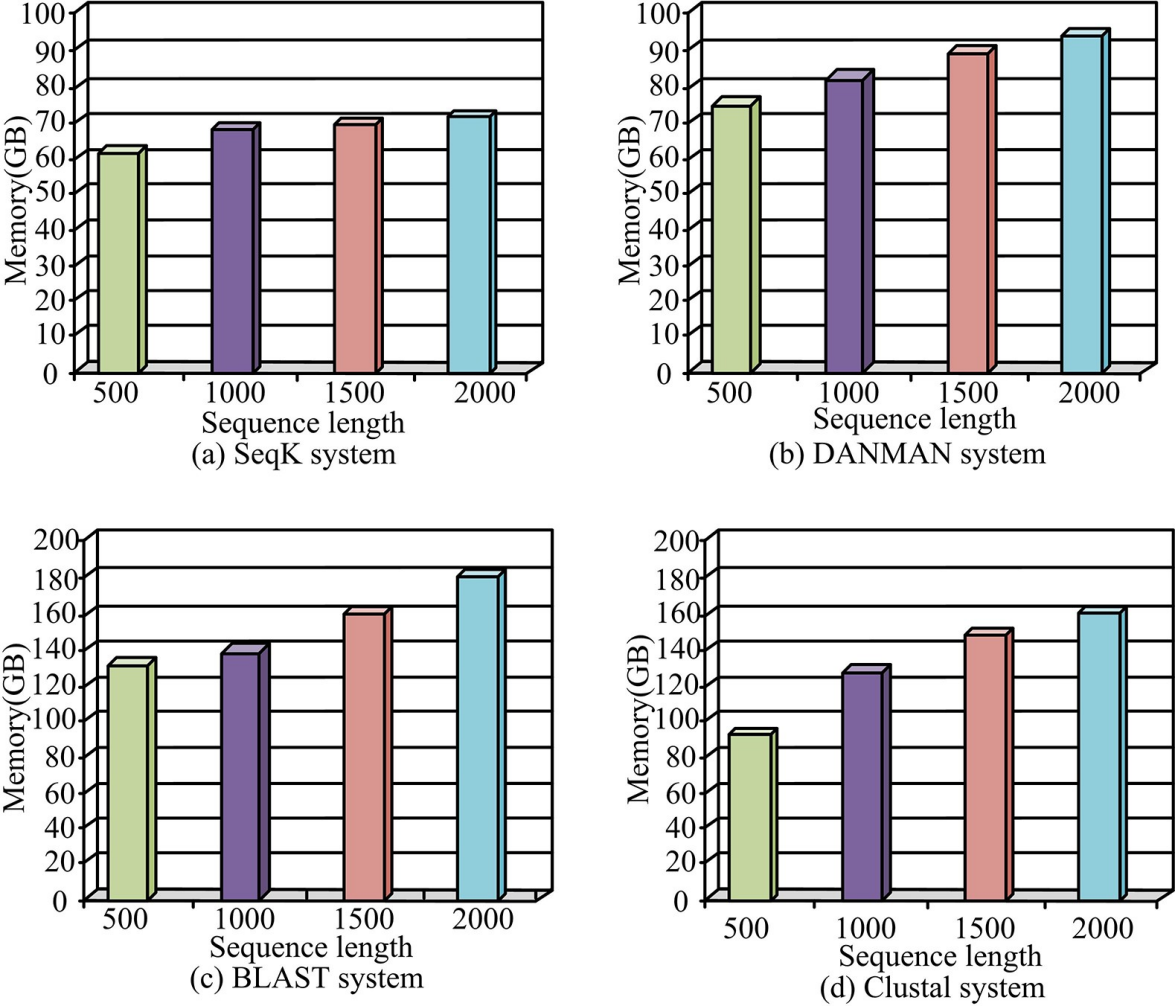
Operational memory under different sequence alignment systems.

Fig 10 shows the operation memory under different sequence alignment systems. Among them, SeqK, danman, blast, and Clustal are used in Fig 10(A)–10(D), respectively. It can be seen that as the length of the processing sequence increased, the running memory of different systems increased by different extents. However, the SeqK system proposed in the study had higher data inclusiveness, so among the four systems, the SeqK system had the smallest amount of running memory, followed by the danman multiple sequence alignment system with good adaptability, then the Clustal system, and finally the blast system. Because the blast system was more suitable for short sequence analysis, when the sequence length was long, the system had a high load, resulting in high running memory. When the length of the sequence was 500, 1000, 1500, and 2000, the SeqK system can reach 62, 68, 69 and 71 GB of running memory, respectively. When the sequence length was 500, 1000, 1500, and 2000, the DANMAN multi-sequence alignment system achieved 74, 82, 88, and 92GB of running memory, respectively. The BLAST system achieved running memory of 127, 140, 160, and 180GB, respectively. The Cluster system achieved 91, 123, 142, and 160GB of running memory, respectively. Therefore, the SeqK system studied and designed had better operation performance.

**Fig 11:**
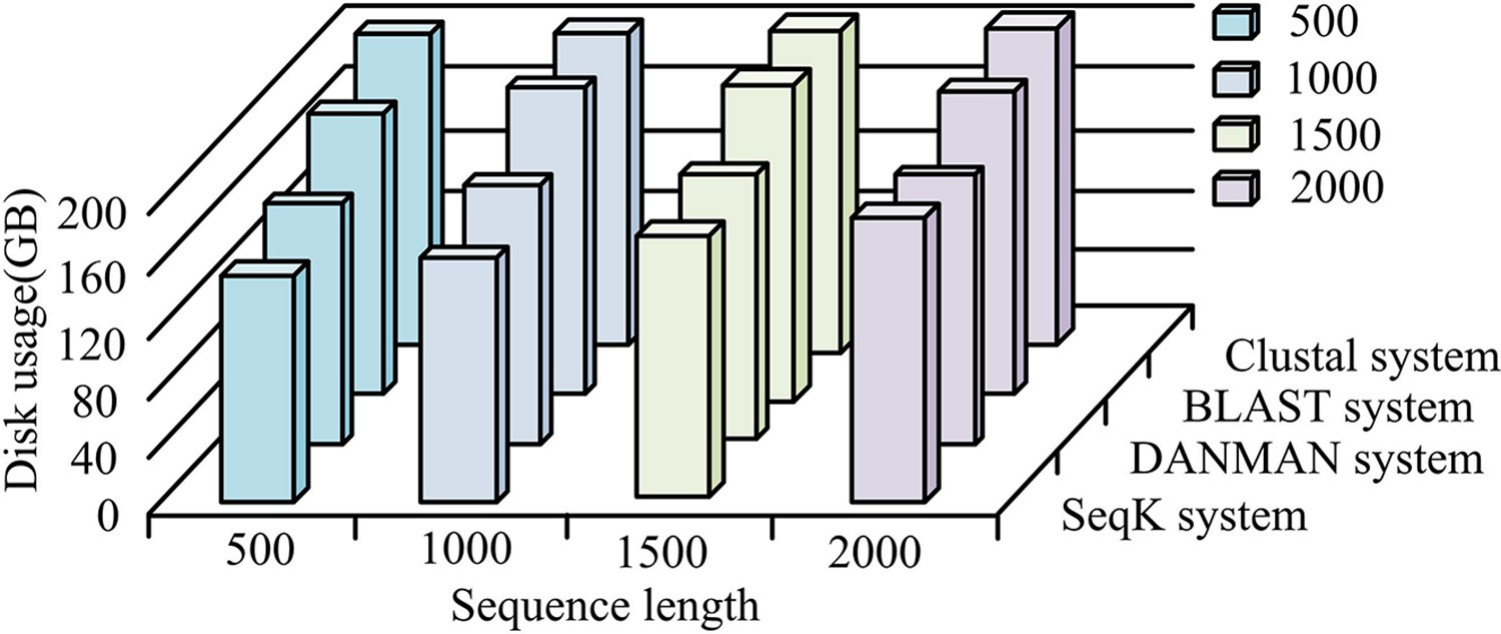
Disk storage under different sequence alignment systems.

Fig 11 shows the disk occupancy under different sequence alignment systems. It can be seen that as the sequence length increased, the disk storage of different systems was also gradually increasing. Among them, the SeqK system had a lower proportion of disks, which was because the SeqK system converted sequences into data of the same frequency according to a unified measure in the process of sequence alignment, so as to reduce the operation process of data, making the whole sequence alignment process more concise, and thus have a lower proportion of disks. The higher proportion of disks in the Clustal system was because the system was a progressive comparison method, and the calculation process was more complex, so the proportion of disks was higher. When the alignment sequence length was 2000, the disk storage of the SeqK system, DANMAN multi-sequence alignment system, Cluster system, and BLAST system was 135GB, 144GB, 187GB, and 152GB, respectively. Therefore, the SeqK system studied this time had a lower disk footprint.

**Fig 12:**
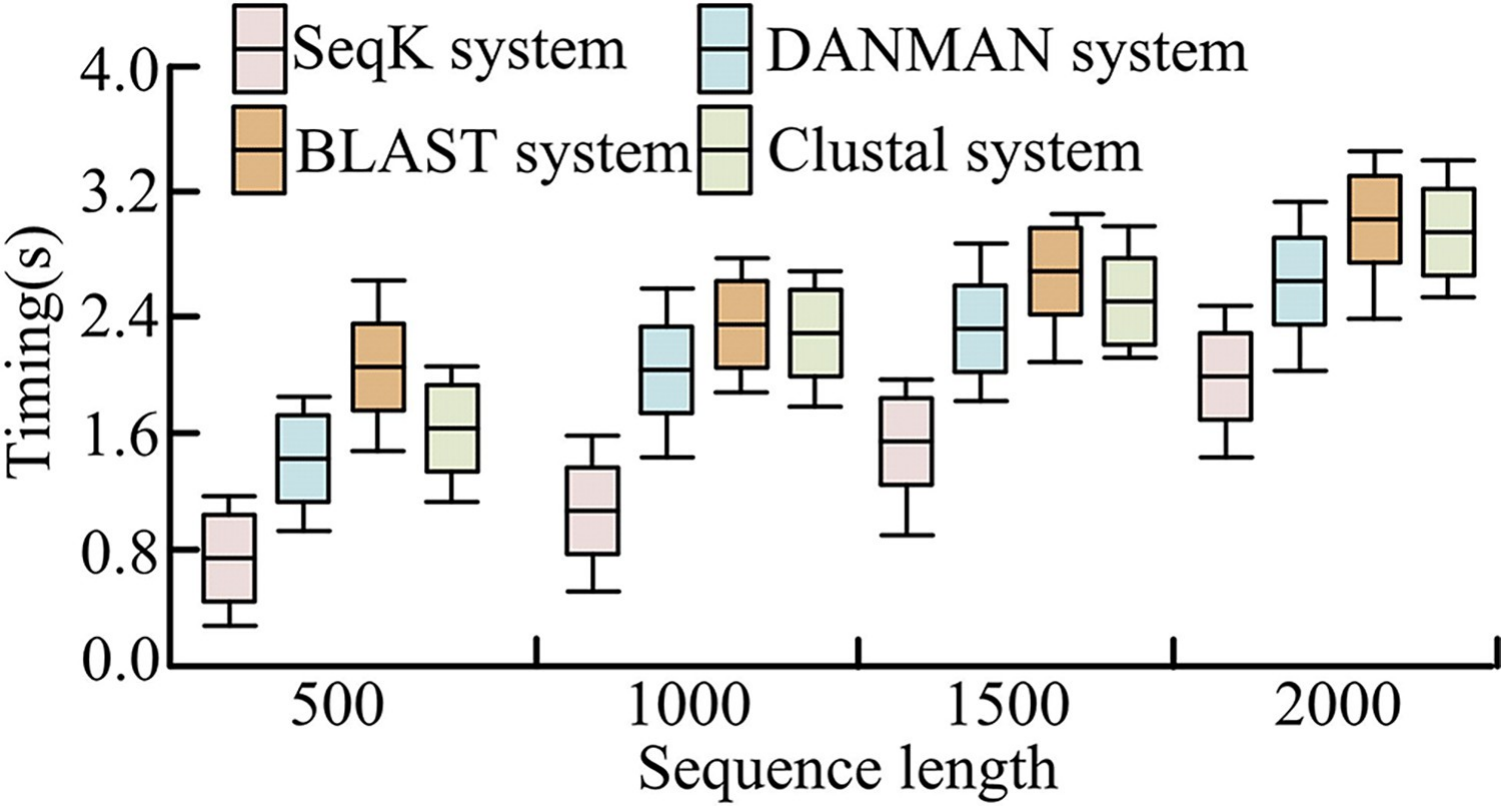
Operation time of different sequence alignment systems.

Fig 12 shows the operation time of different sequence alignment systems. It can be seen that the SeqK system had a shorter operation time among the four-length sequence alignments, and the blast system had the longest operation time. Because the SeqK system can convert sequence data into visual data with the same metric, it improved the operation efficiency. The blast system was limited to the alignment of short sequences, so in the process of alignment, it increased the time of cutting long sequences into short sequences, resulting in the overall operation time being too long. As a progressive comparison system, the Clustal system had a more complex calculation process, so the calculation time of Clustal system was also longer. Because the SeqK system controlled the measurement of operation, its operation efficiency was slightly higher than that of the DANMAN system. When the running sequence length was 2000, the operation time of the SeqK system, DANMAN multi-sequence alignment system, Cluster system, and BLAST system was 1.6s, 2.1s, 2.8s, and 2.6s, respectively. Therefore, the SeqK system had higher sequence alignment efficiency.

## 5 Conclusion

Because gene sequences contain a large amount of biological genetic information, it is of great significance to compare and analyze gene sequences measured by high-throughput sequencing methods. However, the method of gene sequence alignment has not kept pace with the development of sequencing technology, so the analysis of longer gene sequences has become a research difficulty. In order to improve the ability of alignment analysis of long gene sequences, this study proposed to build a statistical alignment model using the k-mer statistic method with high efficiency and low storage requirements. The *D*_2_ series method of k-mer statistic method is used to build the model. Firstly, the gene sequence is segmented, and then the dissimilarity of the segmented sequence is calculated, and the comparison of statistics is completed according to the dissimilarity. At the same time, the SeqK system is also designed to implement the comparison model. The results showed that the model achieved the statistical power of 0.87–0.95 when the sequence coverage was 75%. When *k* was 5, the highest statistical power reached 0.93 in the single-leaf plant sequence dataset. When the cutting length was 2000, the model still achieved a statistical power of 0.93 in the single-leaf plant sequence dataset. The designed system achieved 71 GB of running memory, 135 GB of disk memory, and about 1.8s of running time when the sequence length was 2000. Therefore, the comparison model and system constructed by k-mer statistics have relatively excellent application performance. However, the statistical power of the proposed model in different gene sequence data has certain differences, indicating that the universality of the model still has room to improve, and the model can be improved for the expansion of the scope of application.

## Supporting information

S1 Data (DOC)
