# Supplementary material for: Gene sequence analysis model construction based on k-mer statistics
Source: PLoS One. 2024 Sep 12;19(9):e0306480. doi: 10.1371/journal.pone.0306480 (PMC11392344; doi:10.1371/journal.pone.0306480)
Supplement: S1 Data — (DOC) [file pone.0306480.s001.doc]

**The data in Fig. 6**

| Statistical efficacy P-value | | Sequence length | | | | | | | | | |
| --- | --- | --- | --- | --- | --- | --- | --- | --- | --- | --- | --- |
|  |  | 500 | 1000 | 1500 | 2000 | 2500 | 3000 | 3500 | 4000 | 4500 | 5000 |
| Homo sapiens | r=25% | 0.18 | 0.21 | 0.28 | 0.32 | 0.35 | 0.39 | 0.42 | 0.43 | 0.44 | 0.3 |
|  | r=50% | 0.88 | 0.88 | 0.89 | 0.89 | 0.88 | 0.89 | 0.88 | 0.89 | 0.88 | 0.89 |
|  | r=75% | 0.96 | 0.95 | 0.95 | 0.96 | 0.95 | 0.96 | 0.96 | 0.96 | 0.96 | 0.95 |
| Triticum urartu | r=25% | 0.19 | 0.19 | 0.18 | 0.19 | 0.18 | 0.19 | 0.19 | 0.18 | 0.19 | 0.18 |
|  | r=50% | 0.80 | 0.80 | 0.80 | 0.80 | 0.80 | 0.80 | 0.80 | 0.81 | 0.80 | 0.80 |
|  | r=75% | 0.91 | 0.91 | 0.91 | 0.92 | 0.91 | 0.91 | 0.91 | 0.91 | 0.92 | 0.92 |
| Tupaia chinensis | r=25% | 0.20 | 0.19 | 0.20 | 0.19 | 0.20 | 0.20 | 0.18 | 0.20 | 0.19 | 0.19 |
|  | r=50% | 0.80 | 0.80 | 0.80 | 0.81 | 0.80 | 0.81 | 0.80 | 0.80 | 0.80 | 0.80 |
|  | r=75% | 0.90 | 0.90 | 0.89 | 0.90 | 0.89 | 0.90 | 0.89 | 0.90 | 0.89 | 0.90 |
| Capsicum annuum | r=25% | 0.20 | 0.19 | 0.20 | 0.20 | 0.19 | 0.20 | 0.21 | 0.21 | 0.19 | 0.19 |
|  | r=50% | 0.78 | 0.77 | 0.78 | 0.78 | 0.78 | 0.78 | 0.78 | 0.77 | 0.78 | 0.78 |
|  | r=75% | 0.90 | 0.90 | 0.90 | 0.89 | 0.90 | 0.89 | 0.90 | 0.89 | 0.89 | 0.89 |

**The data in Fig. 7**

| Statistical efficacy P-value | | Coverage rate | | | | |
| --- | --- | --- | --- | --- | --- | --- |
|  |  | 0.2 | 0.4 | 0.6 | 0.8 | 1.0 |
| Homo sapiens | k=5 | 0.62 | 0.80 | 0.88 | 0.89 | 0.89 |
|  | k=6 | 0.78 | 0.81 | 0.81 | 0.82 | 0.82 |
|  | k=7 | 0.41 | 0.59 | 0.71 | 0.79 | 0.82 |
|  | k=8 | 0.22 | 0.37 | 0.45 | 0.56 | 0.59 |
| Triticum urartu | k=5 | 0.81 | 0.92 | 0.92 | 0.93 | 0.93 |
|  | k=6 | 0.82 | 0.85 | 0.85 | 0.85 | 0.85 |
|  | k=7 | 0.71 | 0.74 | 0.77 | 0.79 | 0.79 |
|  | k=8 | 0.39 | 0.58 | 0.71 | 0.75 | 0.78 |
| Tupaia chinensis | k=5 | 0.55 | 0.62 | 0.71 | 0.81 | 0.86 |
|  | k=6 | 0.59 | 0.77 | 0.78 | 0.78 | 0.78 |
|  | k=7 | 0.40 | 0.41 | 0.40 | 0.41 | 0.41 |
|  | k=8 | 0.19 | 0.29 | 0.31 | 0.37 | 0.38 |
| Capsicum annuum | k=5 | 0.69 | 0.81 | 0.82 | 0.84 | 0.84 |
|  | k=6 | 0.54 | 0.76 | 0.78 | 0.80 | 0.82 |
|  | k=7 | 0.57 | 0.61 | 0.63 | 0.66 | 0.68 |
|  | k=8 | 0.28 | 0.43 | 0.53 | 0.55 | 0.57 |

**The data in Fig. 8**

| Statistical efficacy P-value | | Coverage rate | | | | |
| --- | --- | --- | --- | --- | --- | --- |
|  |  | 0.2 | 0.4 | 0.6 | 0.8 | 1.0 |
| Homo sapiens | T=500 | 0.33 | 0.41 | 0.50 | 0.61 | 0.72 |
|  | T=1000 | 0.41 | 0.48 | 0.53 | 0.63 | 0.76 |
|  | T=1500 | 0.48 | 0.51 | 0.58 | 0.68 | 0.84 |
|  | T=2000 | 0.56 | 0.62 | 0.71 | 0.75 | 0.87 |
| Triticum urartu | T=500 | 0.38 | 0.41 | 0.48 | 0.61 | 0.72 |
|  | T=1000 | 0.39 | 0.43 | 0.49 | 0.62 | 0.67 |
|  | T=1500 | 0.49 | 0.58 | 0.67 | 0.78 | 0.82 |
|  | T=2000 | 0.58 | 0.61 | 0.68 | 0.81 | 0.93 |
| Tupaia chinensis | T=500 | 0.18 | 0.24 | 0.29 | 0.38 | 0.52 |
|  | T=1000 | 0.22 | 0.31 | 0.33 | 0.43 | 0.53 |
|  | T=1500 | 0.28 | 0.38 | 0.48 | 0.61 | 0.75 |
|  | T=2000 | 0.31 | 0.42 | 0.56 | 0.63 | 0.80 |
| Capsicum annuum | T=500 | 0.18 | 0.23 | 0.29 | 0.47 | 0.52 |
|  | T=1000 | 0.22 | 0.29 | 0.36 | 0.52 | 0.54 |
|  | T=1500 | 0.31 | 0.35 | 0.47 | 0.61 | 0.74 |
|  | T=2000 | 0.41 | 0.48 | 0.59 | 0.65 | 0.79 |

**The data in Fig. 9**

| Statistical efficacy P-value | | Coverage rate | | | | |
| --- | --- | --- | --- | --- | --- | --- |
|  |  | 0.2 | 0.4 | 0.6 | 0.8 | 1.0 |
| Homo sapiens | L=5 | 0.78 | 0.79 | 0.80 | 0.80 | 0.80 |
|  | L=6 | 0.61 | 0.71 | 0.73 | 0.75 | 0.78 |
|  | L=7 | 0.65 | 0.69 | 0.71 | 0.71 | 0.71 |
|  | L=8 | 0.40 | 0.59 | 0.65 | 0.69 | 0.70 |
| Triticum urartu | L=5 | 0.80 | 0.81 | 0.81 | 0.82 | 0.82 |
|  | L=6 | 0.78 | 0.79 | 0.79 | 0.81 | 0.81 |
|  | L=7 | 0.48 | 0.71 | 0.72 | 0.72 | 0.72 |
|  | L=8 | 0.67 | 0.69 | 0.70 | 0.70 | 0.70 |
| Tupaia chinensis | L=5 | 0.68 | 0.79 | 0.79 | 0.81 | 0.81 |
|  | L=6 | 0.51 | 0.59 | 0.71 | 0.72 | 0.73 |
|  | L=7 | 0.58 | 0.66 | 0.68 | 0.70 | 0.71 |
|  | L=8 | 0.38 | 0.51 | 0.57 | 0.58 | 0.59 |
| Capsicum annuum | L=5 | 0.71 | 0.78 | 0.81 | 0.81 | 0.81 |
|  | L=6 | 0.63 | 0.74 | 0.75 | 0.76 | 0.76 |
|  | L=7 | 0.44 | 0.63 | 0.69 | 0.71 | 0.73 |
|  | L=8 | 0.58 | 0.59 | 0.61 | 0.61 | 0.62 |

**The data in Fig. 10**

| System | Sequence length | Memory (GB) |
| --- | --- | --- |
| SeqK system | 500 | 62 |
|  | 1000 | 68 |
|  | 1500 | 69 |
|  | 2000 | 71 |
| DANMAN system | 500 | 74 |
|  | 1000 | 82 |
|  | 1500 | 88 |
|  | 2000 | 92 |
| BLAST system | 500 | 127 |
|  | 1000 | 140 |
|  | 1500 | 160 |
|  | 2000 | 180 |
| Clustal system | 500 | 91 |
|  | 1000 | 123 |
|  | 1500 | 142 |
|  | 2000 | 160 |

**The data in Fig. 11**

| System | Sequence length | Disk usage (GB) |
| --- | --- | --- |
| SeqK system | 500 | 91 |
|  | 1000 | 107 |
|  | 1500 | 118 |
|  | 2000 | 135 |
| DANMAN system | 500 | 94 |
|  | 1000 | 112 |
|  | 1500 | 125 |
|  | 2000 | 144 |
| BLAST system | 500 | 98 |
|  | 1000 | 119 |
|  | 1500 | 137 |
|  | 2000 | 152 |
| Clustal system | 500 | 108 |
|  | 1000 | 124 |
|  | 1500 | 157 |
|  | 2000 | 187 |

**The data in Fig. 12**

| System | Sequence length | Timing(s) |
| --- | --- | --- |
| SeqK system | 500 | 0.7 |
|  | 1000 | 1.0 |
|  | 1500 | 1.4 |
|  | 2000 | 1.6 |
| DANMAN system | 500 | 1.4 |
|  | 1000 | 1.9 |
|  | 1500 | 2.0 |
|  | 2000 | 2.1 |
| BLAST system | 500 | 2.0 |
|  | 1000 | 2.2 |
|  | 1500 | 2.4 |
|  | 2000 | 2.6 |
| Clustal system | 500 | 1.7 |
|  | 1000 | 2.1 |
|  | 1500 | 2.3 |
|  | 2000 | 2.8 |
